# Supplementary material for: The Urinary Metabolomic Fingerprint in Extremely Preterm Infants on Total Parenteral Nutrition vs. Enteral Feeds
Source: Metabolites. 2023 Aug 24;13(9):971. doi: 10.3390/metabo13090971 (PMC10537655; doi:10.3390/metabo13090971)
Supplement: Supplementary file 1 [file metabolites-13-00971-s001.zip › Figures S1-S3.pdf]

metabolites

MDPI

Supplementary Materials

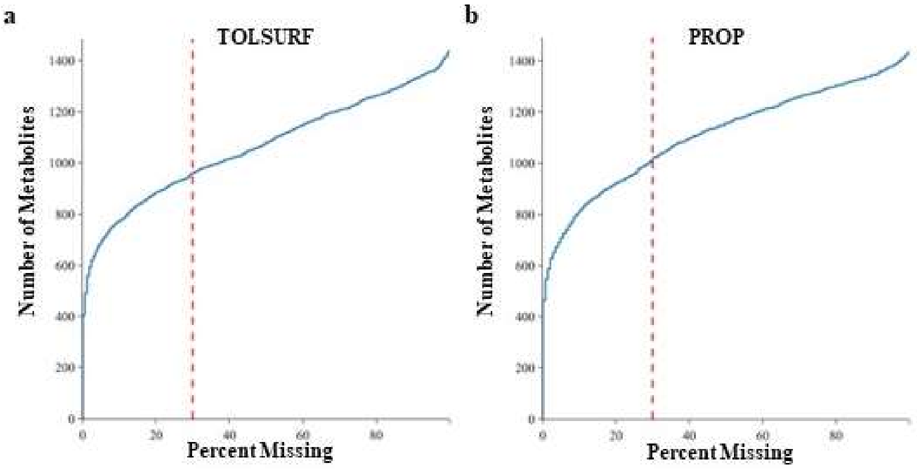

Figure S1. Empirical distribution of missing data across all detected metabolites. **(a)** TOLSURF cohort, **(b)** PROP cohort. Metabolites with < 30% missing data are shown (dashed red line).

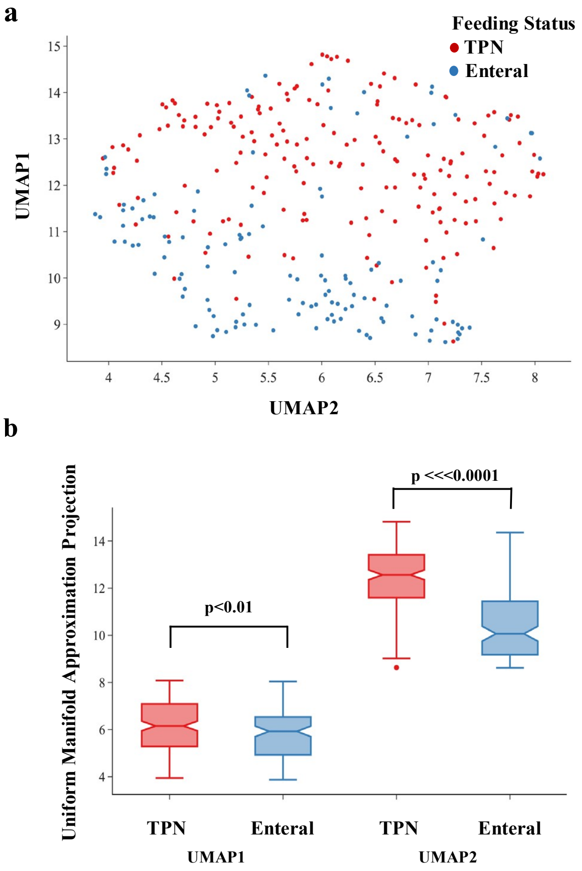

Figure S2. UMAP of 913 biochemicals identified in the urine of infants from the TOLSURF and PROP cohorts collected between 23–30 days of life. **(a)** UMAP shows a visual separation of infants based on feeding status. **(b)** UMAP 1st latent dimension showed a nominal yet significant difference

between feeding status ($p = 0.0027$) while UMAP 2nd dimension showed a strong statistical difference ($p = 8.64 \times 10^{-24}$).

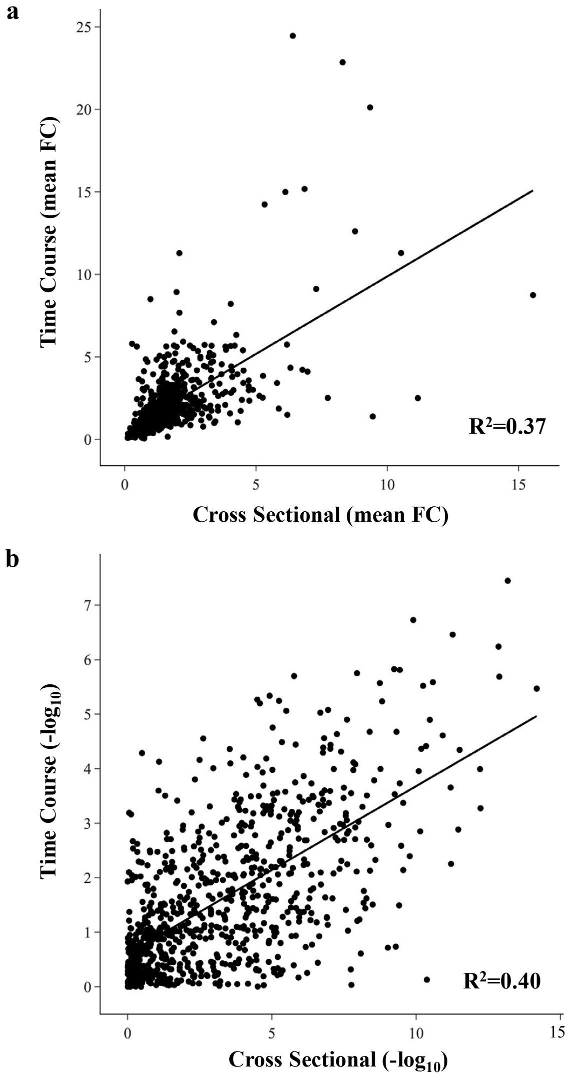

Figure S3. Regression analysis of fold change and *p*-value between cross-sectional and longitudinal analyses. **(a)** The fold change for longitudinal data in the TOLSURF cohort is determined by comparing the metabolomic profiling results taken five days before TPN was completely discontinued and 0–2 days after discontinuation. Within the cross-sectional analysis, the fold change is calculated by averaging the results from the TOLSURF and PROP cohorts. **(b)** The *p*-values obtained from the analyses are transformed using a negative logarithm to the base ten scale (negative log10 scale).
